# Simultaneous Pancreas-Kidney Transplantation Vs. Deceased Donor Kidney Transplantation in Patients With Diabetes Mellitus – An Ongoing Controversy

**DOI:** 10.3389/ti.2025.15867

**Published:** 2025-12-30

**Authors:** A. C. Gruessner, R. W. G. Gruessner

**Affiliations:** 1 SUNY Downstate Health Sciences University Department of Medicine, New York, NY, United States; 2 SUNY Downstate Health Sciences University College of Medicine, New York, NY, United States

**Keywords:** pancreas transplant, kidney transplant, simultaneous kidney pancreas transplantation, outcome, mortality

The debate about the benefits of simultaneous pancreas-kidney transplantation (SPK) versus deceased donor kidney transplantation alone (DDKTA) has persisted since the inception of SPK in 1966 [1].

In this issue of *Transplant International*, the study entitled “*Reassessing Simultaneous Pancreas-Kidney* vs*. Kidney Transplant Alone: A propensity-weighted Analysis of Survival and Morbidity*” seeks to re-address this topic through a retrospective registry (SRTR) analysis. The authors use this type of analysis to produce real-world evidence to support clinical policy and decision making.

In their study, the authors challenge the survival benefit for all diabetes types for SPK vs. DDKTA and, consequently, question any prioritization for SPK. However, their conclusions are problematic due to the nature of their study design and methodology.

Randomized controlled trials (RCT) are the gold standard for establishing causal relationships. Unfortunately, RCTs in the context of SPK vs. DDKTA are deemed infeasible due to ethical concerns. If a qualified SPK candidate wishes to undergo a simultaneous transplant to become not only dialysis-free but also diabetes-free, why should this candidate be denied an SPK based solely on a randomization protocol that would give him/her only a 50% vs. a 100% chance to become insulin-independent?

Due to the infeasibility of a definitive RCT, the authors employed a propensity-weighted analysis. Undoubtedly, this is a sophisticated statistical method designed to simulate the balance of an RCT by adjusting for confounding variables. However, propensity-weighted analysis should only be performed if all relevant factors are available.

Hence, the central question is: are the SPK and DDKTA groups in this study truly comparable? As evidenced in Table 1 of this article, the two groups differ significantly across all included baseline characteristics. This imbalance raises concerns about residual confounding and the validity of direct outcome comparisons, even after sophisticated statistical adjustment.

The majority of SPK recipients had type 1 diabetes and met stringent listing criteria for pancreas transplantation. In contrast, DDKTA recipients either did not qualify, were declined, or opted out of a simultaneous pancreas transplant. For this reason alone, the two groups represent fundamentally different populations. The significant disparities in donor and recipient characteristics, as well as the unweighted survival outcomes, further underscore this deficiency. Therefore, applying weighted propensity scoring to estimate the probability of receiving an SPK among DDKTA recipients introduces bias, as the counterfactual scenario is not clinically feasible for most candidates. This methodological shortfall may cause misrepresentation of this study’s true comparative effectiveness of SPK vs. DDKTA and will lead to wrong conclusions [2].

The study’s overall comparative analysis between SPK and DDKTA recipients is challenged by significant cohort imbalances. There are fewer SPK recipients, and they have predominantly type 1 diabetes, whereas DDKTA recipients are more numerous and more often have type 2 diabetes. This disparity introduces inherent confounding, as the diabetes type is closely linked to disease progression, secondary complications, comorbidity profiles, and transplant eligibility.

Moreover, several potentially influential patient characteristics were not accounted for in the authors’ analysis, including age at disease onset, duration of diabetes, severity of disease and the severity of secondary complications, regional variations in treatment practices as well as transplant center volume. Almost all transplant centers in the United States transplant kidneys but not all centers transplant pancreata. These variables may have influenced both treatment selection and outcomes, further complicating interpretation of the study’s results using incomplete registry data.

Statistical adjustments cannot fully mitigate these confounders, especially when donor quality and transplant criteria vary across groups. As the authors showed, a more robust approach with stratified analyses within homogenous subgroups—such as limiting comparisons to patients with type 1 diabetes to reduce heterogeneity and enhance interpretability -- revealed a lower mortality rate for SPK recipients in this study. This result was confirmed by previous studies which evaluated highly selective cohorts specifically for patients stratified either for type 1 or type 2 diabetes. The reason for these stratifications was to assess the differences in those groups and minimize the selection bias [3].

An SPK transplant is clearly a more difficult procedure than a DDKTA since two organs are transplanted. This naturally carries a higher risk of surgical morbidity and graft rejection. Hence, this finding in the present study was to be expected. Yet importantly, it did not have an impact on patient and kidney graft survival.

The authors challenge the overall benefit of an SPK in general but do not take into consideration that the patient who only receives a DDKTA remains diabetic. After all, diabetes is the main reason for their end-stage renal disease. The DDKTA transplant is only a treatment for one secondary diabetic complication but not for the underlying reason of the patient’s end-stage kidney disease. Post transplant, SPK recipients are usually completely off insulin while DDKTA recipients must continue insulin treatment [4]. While both SPK and DDKTA recipients require lifelong maintenance immunosuppression, the impact on diabetes management diverges significantly between the two. In DDKTA recipients, immunosuppressive agents - especially corticosteroids and calcineurin inhibitors – can worsen insulin resistance, exacerbate hyperglycemia, and complicate insulin dosing and glucose monitoring.

As there has been mounting evidence in the literature that diabetic complications in an SPK recipient with a functioning pancreas transplant can be halted, improved or even reversed, this severe disease is ongoing in the DDKTA recipient and, as mentioned, will be even harder to manage due to the side effects of immunosuppression. Cardiac disease, retinopathy, and neuropathy continue to progress and may get worse in DDKTA recipients. There is no question that early restoration of kidney function is essential for patient survival. Yet the real impact of a functioning pancreas graft can only be detected after several years as demonstrated in various studies [5].

The authors also question the advantages of improvement in quality of life for SPK recipients. However, there is a plethora of evidence in the literature demonstrating more significant quality of life improvement after SPK vs. DDKTA [6].

For labile diabetic patients, it is important to emphasize that SPK still remains the best treatment option to become fully insulin independent [4]. It is now apparent that despite great technological improvements through smart pumps, artificial pancreas and other forms of beta-call replacement therapy such as islet and stem cell transplants, these modalities do not consistently result in total freedom from insulin injections. Hence, the currently available new technologies along with DDKTA do not substitute for an SPK as patient surveys have shown [7].

In their summary, the authors challenge listing of any qualified diabetic and uremic patient for an SPK over a DDKTA. They recommend careful counseling regarding higher morbidity and rejection episodes in SPK recipients. However, even in their methodologically flawed study with a higher early complication rate, it is important to emphasize that the mortality rate in SPK vs. DDKTA was not any higher.

Unfortunately, there is a clear disconnect between the study results and the authors’ conclusions. How can they possibly advocate against SPK if neither graft survival nor mortality was negatively impacted in their own study? It is obvious that the authors completely disregard that an SPK recipient after a successful dual transplant will be entirely insulin-free and enjoy a higher quality of life.

We agree with the authors about appropriate pretransplant counseling, but with one caveat. We feel strongly that qualified SPK candidates should always be referred to centers offering both SPK and DDKTA. Centers with experience in pancreas transplantation will provide better comprehensive patient counseling than centers without experience in pancreas transplantation.

Aside from the fact that in the US only 2.4% of all kidneys in 2024 were used for SPK, a prioritization of an SPK should continue. After all, the present study shows no inferior outcome for patient and kidney graft survival between the two groups.

In summary, the authors bolster distorted conclusions in their potentially influential publication based on a inappropriate study design and methodology. An equally contentious study in 2004 which falsely reported significantly worse patient survival and higher mortality after solitary pancreas transplantation resulted in a marked decline in solitary pancreas transplant activity [8]. A subsequent analysis of the original data revealed substantial flaws in methodology [9, 10]. Although the results and conclusions of the original study were subsequently proven wrong, the later publication with corrected data received far less attention. The damage was done: the field of solitary pancreas transplantation almost vanished [11]. It is our hope that the benefits of an SPK are not judged on the basis of the present publication in *Transplant International*.

## Data Availability

The original contributions presented in the study are included in the article/supplementary material, further inquiries can be directed to the corresponding author.
